# Radiolabeling and evaluation of ^64+67^Cu-2,2’-(1,2,4-Oxadiazole-3,5-diyl)di aniline as a theranostic agent for solid tumors

**DOI:** 10.1038/s41598-025-21804-x

**Published:** 2025-10-27

**Authors:** Mohamed A. Gizawy, Mohammed Salah Ayoup, Ahmad S. Kodous, Mohamed Taha Abdelrahman, M. A. Motaleb

**Affiliations:** 1 Labeled compounds Department, Hot Laboratories Center, Egyptian Atomic Energy Authority, P.O. Box 13759, Cairo, Egypt; 2 Chemistry Department, Faculty of Science, Alexandria University, P.O. Box 426, Ibrahimia, Alexandria Egypt; 3 Radiation Biology Department, National Center for Radiation Research and Technology (NCRRT), Egyptian Atomic Energy Authority (EAEA), P.O. Box 13759, Cairo, Egypt; 4 Radioisotopes Department, Nuclear Research Center, Egyptian Atomic Energy Authority, P.O. Box 13759, Cairo, Egypt

**Keywords:** ^64+67^Cu, Oxadiazole dianiline derivative, Theranostics, EGFR, VEGFR2, Coordination chemistry, Medicinal chemistry, Nuclear chemistry, Drug discovery, Cancer models

## Abstract

Copper-64 (^64^Cu) and Copper-67 (^67^Cu) are promising theranostic isotopes for PET imaging and targeted radiotherapy. In this study, 2,2’-(1,2,4-Oxadiazole-3,5-diyl)di aniline (ODDA) was successfully radiolabeled with ^64+67^Cu, achieving high radiolabeling yield (97.0 ± 0.8%) and excellent stability in serum over five days. The optimized radiolabeling conditions included a ligand concentration of 30 µg, pH 4, a reaction time of 15 min, and a temperature of 60 °C. The biodistribution study in Ehrlich ascites carcinoma-bearing mice showed an initial tumor uptake of 7.8 ± 0.4%ID/g at 0.5 h, peaking at 18.0 ± 0.3ID/g at 2 h, confirming selective tumor accumulation. The log P value of -0.8 indicated a hydrophilic nature, leading to predominant renal clearance. Molecular docking studies showed strong binding to EGFR (-9.1 kcal/mol) and VEGFR2 (-8.6 kcal/mol), suggesting potential anticancer activity.

## Introduction

Copper-64 (^64^Cu) and Copper-67 (^67^Cu) are two important radioisotopes of copper that have garnered significant interest in nuclear medicine and radiopharmaceuticals due to their unique nuclear and chemical properties^1^. ^64^Cu is widely used in PET imaging due to its favorable decay characteristics and ability to provide high-resolution images of tumor metabolism, receptor expression, and angiogenesis. It has a relatively long half-life of 12.7 h compared to other PET radioisotopes, which provide an extended imaging window, allowing for the monitoring of biological processes over time^2^. On the other hand, ^67^Cu is a pure β⁻ emitter with a half-life of approximately 61.8 h, making it highly suitable for targeted radiotherapy. It emits beta particles with a maximum energy of 577 keV, which is effective for inducing cytotoxicity in malignant cells while minimizing damage to surrounding healthy tissue^3,4^.

The chemical versatility of copper allows for the development of stable chelating agents that can be conjugated with a wide range of biologically active molecules, including peptides, antibodies, and small molecules^5,6^. This has paved the way for the design of radiopharmaceuticals that combine high specificity for tumor targets with the therapeutic benefits of radiotherapy^7^.

Oxadiazole derivatives have emerged as versatile and structurally robust scaffolds in medicinal chemistry, owing to their notable thermal and chemical stability, along with a broad spectrum of biological activities^8^. These heterocyclic compounds are frequently incorporated into drug-like molecules, particularly as bioisosteric replacements for esters and amides, due to their comparable electronic properties and ability to engage in hydrogen bonding interactions^9^. Structurally, oxadiazoles are five-membered aromatic rings composed of two nitrogen atoms, one oxygen atom, and two carbon atoms. Based on the relative positions of the heteroatoms, four main regioisomers exist: 1,2,3-; 1,2,4-; 1,2,5-; and 1,3,4-oxadiazoles^10,11^. Among these, the 1,2,4-oxadiazole subclass has drawn particular interest due to its well-documented pharmacological potential, including anticancer, antioxidant, anti-inflammatory, analgesic, and neuroprotective effects^12–24^.

The growth factors EGFR and VEGFR2 (epidermal growth factor receptor and vascular endothelial growth factor receptor 2) play significant roles in the genesis and progression of cancer. These receptors overexpression or mutation may result in uncontrolled cell proliferation, angiogenesis, and metastasis. As a consequence, tailored medicines that precisely block EGFR and VEGFR2 have been developed to treat some forms of cancer, including non-small cell lung cancer and colorectal cancer^25–28^. These medications reduce tumor development and improve patient outcomes by inhibiting the signaling pathways that these receptors trigger. However, it is vital to highlight that the efficacy of these targeted medicines may vary depending on the patient^29,30^. EGFR and VEGFR signaling pathways enhance tumour development and angiogenesis via substantial autocrine and paracrine interaction^31^. Cells release extracellular ligands that bind to receptors on identical cells to begin signal transduction in autocrine signalling, whereas growth factors from one cell target neighbouring cells to activate signalling cascade in paracrine signalling. VEGF-A triggers Erk and Akt signalling and promotes breast cancer cell proliferation and survival through autocrine signalling^32^. EGFR pathway activation also enhances tumour cell VEGF-A production, which interacts with VEGFR2 on endothelial cells to promote paracrine proliferation, migration, and differentiation^33^.

This study aims to develop and evaluate a novel theranostic agent based on the radiolabeling of 2,2'-(1,2,4-Oxadiazole-3,5-diyl)di aniline (ODDA) with ^64+67^Cu radioisotopes. The work focuses on optimizing the radiolabeling conditions, assessing the stability of the radiolabeled complex, and investigating its biodistribution in tumor-bearing mice. Additionally, molecular docking studies were performed to explore the binding interactions of the complex with EGFR and VEGFR2, two key receptors implicated in tumor proliferation and angiogenesis.

## Experimental

### Chemicals, reagents and instrumentation

In this study, all chemicals and reagents were carefully selected to ensure the highest purity and reliability, with each compound being of high analytical grade to guarantee accuracy and reproducibility in all experimental procedures. Gamma ray radioactivity levels were determined with a NaI(Tl) scintillation counter (SR7 model, England).

### Synthesis of 2,2'-(1,2,4-oxadiazole-3,5-diyl) di aniline (ODDA)

The ODDA ligand was prepared according to the method reported by Ayoup et al.^34^.

### Production of ^64+67^Cu radioisotopes

About 100 mg of a natural Zn(II) target was exposed to neutron radiation for 24 h at the irradiation grid around the core of the Egyptian second research reactor (ETRR-2), Inshas, Egypt. After five days of decay, the irradiated zinc was dissolved in a solution of HCl with the pH adjusted to 3. The resulting solution underwent dynamic radiochemical separation by applying the method described in our earlier study^35^. Finally, the eluted fractions were subjected to quality control tests to ensure their suitability for subsequent radiolabeling experiments.

#### Note on isotope usage and notation:

Since both isotopes were used directly in combination for all subsequent labeling steps without physical or chemical separation, the notation (^64+67^Cu) is consistently adopted throughout the manuscript.

### Radiolabeling of ODDA with ^64+67^Cu radioisotopes

The radiolabeling of ODDA with ^64+67^Cu was carried out using a direct complexation method. The effects of ligand amount, medium pH, reaction temperature and incubation time were examined to optimize the radiolabeling conditions. In a 3 ml-borosilicate vial, 10 µL of ^64+67^CuCl_2_ (100 MBq) in 0.1 M HCl was added to a 30 µL of ODDA ligand in ethanol (1 mg/1 mL). 500 µL of sodium acetate buffer with a pH of 4 is then added. The reaction mixture was incubated at 60 °C for 15 min^36,37^.

### Assessment of radiolabeling yield of ^64+67^Cu-ODDA

**Fig. 1:**
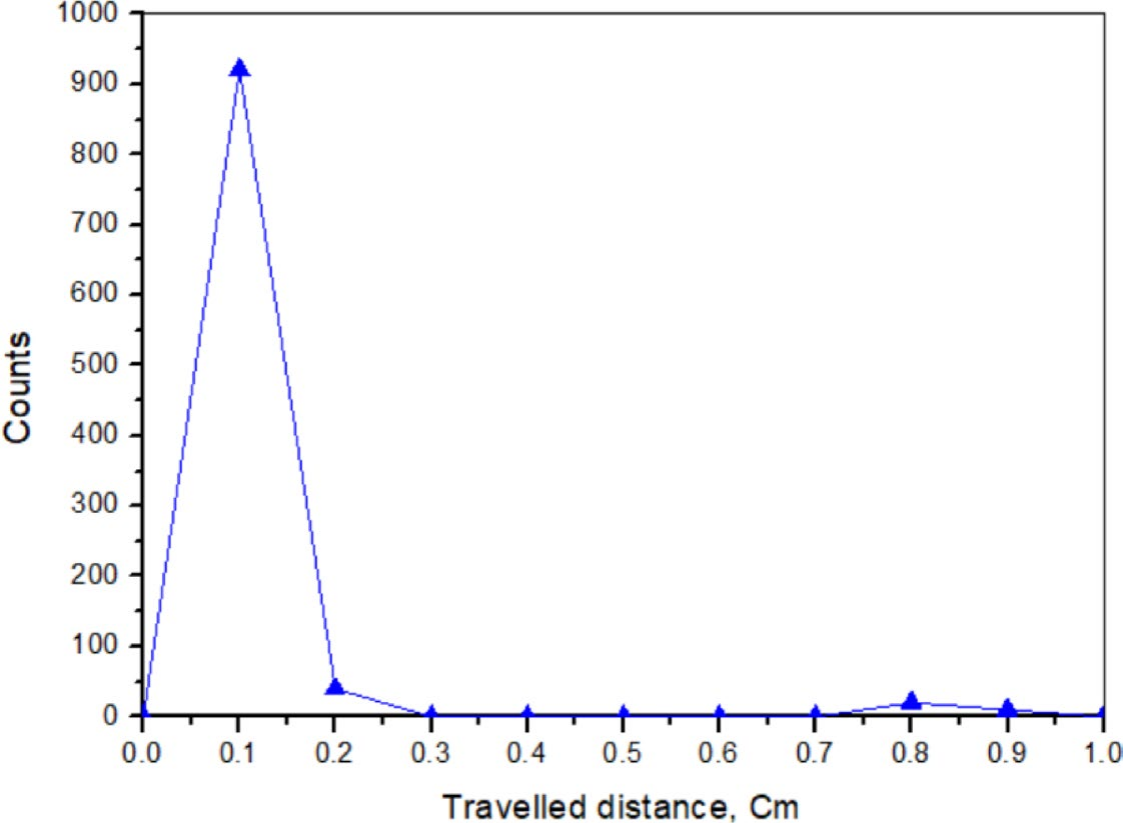
Radio-TLC chromatogram of ^64+67^Cu-ODDA complex using 10% ammonium acetate:methanol (1:1, v/v) as the mobile phase.

The radiolabeling efficiency was assessed using thin-layer chromatography (TLC) with a mobile phase consisting of 10% ammonium acetate: methanol (1:1). In this system, free ^64+67^Cu remains near the origin, while the ^64+67^Cu-ODDA complex moves to a higher R_f_ value^36^. A representative chromatogram is shown in Fig. 1.

### Stability of ^64+67^Cu-ODDA complex in serum

The in-vitro stability of the ^64+67^Cu-ODDA complex was investigated by incubating 500 μL of freshly collected mouse serum with 200 μL of the ^64+67^Cu-ODDA complex (50 MBq) for 5 days at 37 °C. A 10 µL aliquot was taken at different time intervals during incubation and analyzed using TLC^38^.

### Lipophilicity test of the ^64+67^Cu-ODDA complex

The lipophilicity of the ^64+67^Cu-ODDA complex was determined using a partition coefficient (log P) assay. The complex was mixed with equal volumes of 1-octanol and phosphate buffer (0.025 M, pH 7.4) in a centrifuge tube. The mixture was vortexed for 1 min. and then centrifuged at 5000 rpm for 5 min. Samples from the 1-octanol and aqueous layers were counted using a gamma counter to determine the distribution of the complex between the two phases^39^.

### Biodistribution study of the ^64+67^Cu-ODDA complex in tumor bearing mice

All animal experiments were performed in accordance with relevant guidelines and regulations. The experimental protocols were approved by the Animal Ethics Committee of the Egyptian Atomic Energy Authority (EAEA), Cairo, Egypt. The study was carried out in compliance with the ARRIVE guidelines (https://arriveguidelines.org). The in-vivo biodistribution study was conducted using male Swiss albino mice which were seven to eight weeks old, weighing between 25 and 30 g, procured from the animal house of the Egyptian Atomic Energy Authority and acclimatized under standard laboratory conditions with free access to food and water. To conduct the solid tumor transplant, a solution of 2.5 × 10^6^ of Ehrlich ascites carcinoma cells (EAC) was injected intramuscularly into the right thigh of each mouse, while the left thigh served as a control^40^. Each solid tumor-bearing mouse received an intravenous injection of 0.2 ml of ^64+67^Cu-ODDA complex (50 MBq). At 0.5, 1, 2, 4, 12, and 24 h post-injection (p.i.), each mouse was anesthetized using an intraperitoneal injection of a ketamine/xylazine combination (ketamine: 75–100 mg/kg; xylazine: 5–10 mg/kg). After confirming deep anesthesia, euthanasia was performed by cervical dislocation. The organs were then collected and counted using a γ-ray scintillation counter. For every sample, the following formula was used to determine the amount of the administered dose per gram of tissue or organ (% ID/g)^38^.

$$\begin{aligned} \% {\text{ID/g}}= \frac{{{\text{Activity of tissue or fluid or organ}} \times 100}}{{{\text{Total injected activity}} \times {\text{Weight of tissue or fluid or organ}}}} \end{aligned}$$

### Molecular docking

Docking computations were performed using the Auto Dock 4.2 module with Gasteiger’s partial charges attached to atoms of designed drug ligands programe. Calculations were achieved with ligand–protein patterns. Non-polar hydrogen atoms were joined together to explain rotatable bonds. Kollman fused atomic charges with recovery parameters were calculated after adding central hydrogen atoms using AutoDock^41^. Vanderwaal and electrostatic bonds were determined using parameter set- and dielectric distance-dependent functions, respectively. Simulative docking was performed using the Solis and Wets local procedure and the Lamarck genetic algorithm. Ligand molecules were identified in initial positions, orientations and torsions. Upon docking, all rotatable torsions were removed. Each docking experiment was blocked following an ultimate energy estimate of 250,000 in 10 separate runs^42^.

## Results and discussion

### Optimization of the radiolabeling conditions of ODDA

The radiolabeling efficiency of 2,2'-(1,2,4-Oxadiazole-3,5-diyl)di aniline with ^64^Cu and ^67^Cu was optimized by systematically investigating key reaction parameters, including ligand concentration, pH, reaction time, and temperature. The optimization aimed to maximize the radiolabeling yield while minimizing the presence of unbound Cu ions^43,44^.

#### Effect of Ligand Concentration

**Fig. 2:**
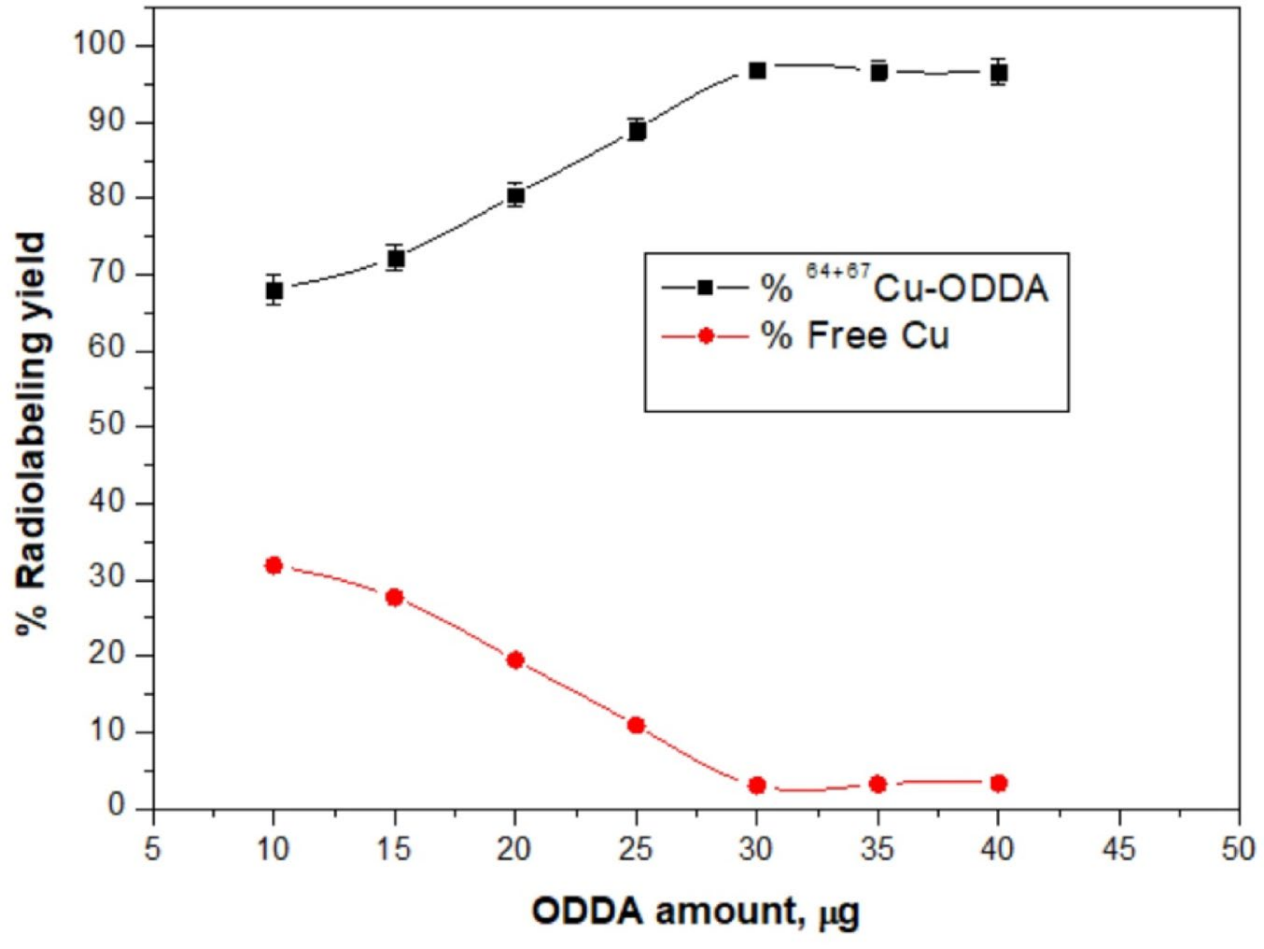
Effect of ligand concentration on radiolabeling efficiency.

To determine the optimal ligand concentration, the radiolabeling reaction was carried out with ligand amounts ranging from 10 to 40 µg. As shown in Fig. 2, increasing the ligand concentration improved radiolabeling efficiency up to 30 µg, where the highest radiolabeling yield of 97.0 ± 0.8% was achieved. Beyond this concentration, no significant improvement was observed, likely due to ligand saturation. Conversely, at ligand amounts below 30 µg, an increased fraction of free Cu ions was detected, indicating insufficient ligand availability for complete complexation.

#### Effect of pH

**Fig. 3:**
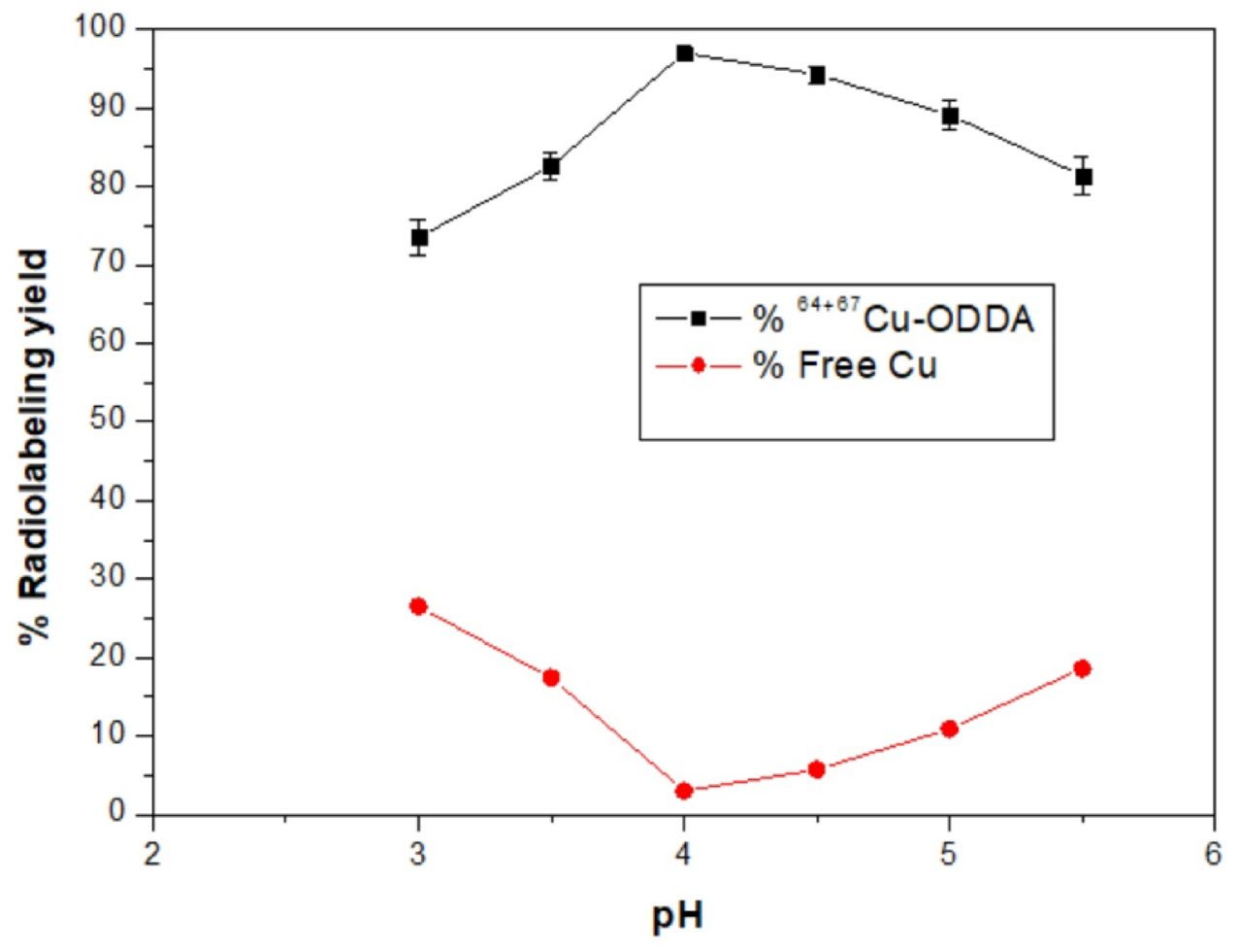
Effect of pH on radiolabeling efficiency.

The influence of pH on radiolabeling efficiency was evaluated over a range of 3.0 to 5.5. Figure 3 clearly illustrates that the optimal radiolabeling yield (97.0 ± 0.8%) was obtained at pH 4. At lower pH values (< 4), reduced labeling efficiency was observed due to proton competition at coordination sites, leading to incomplete complexation. At higher pH (> 4), hydrolysis of Cu(II) ions resulted in colloidal precipitation, reducing the labeling yield. These findings align with previous studies on copper radiolabeling chemistry, emphasizing the importance of maintaining slightly acidic conditions for optimal coordination.

#### Effect of reaction time

**Fig. 4:**
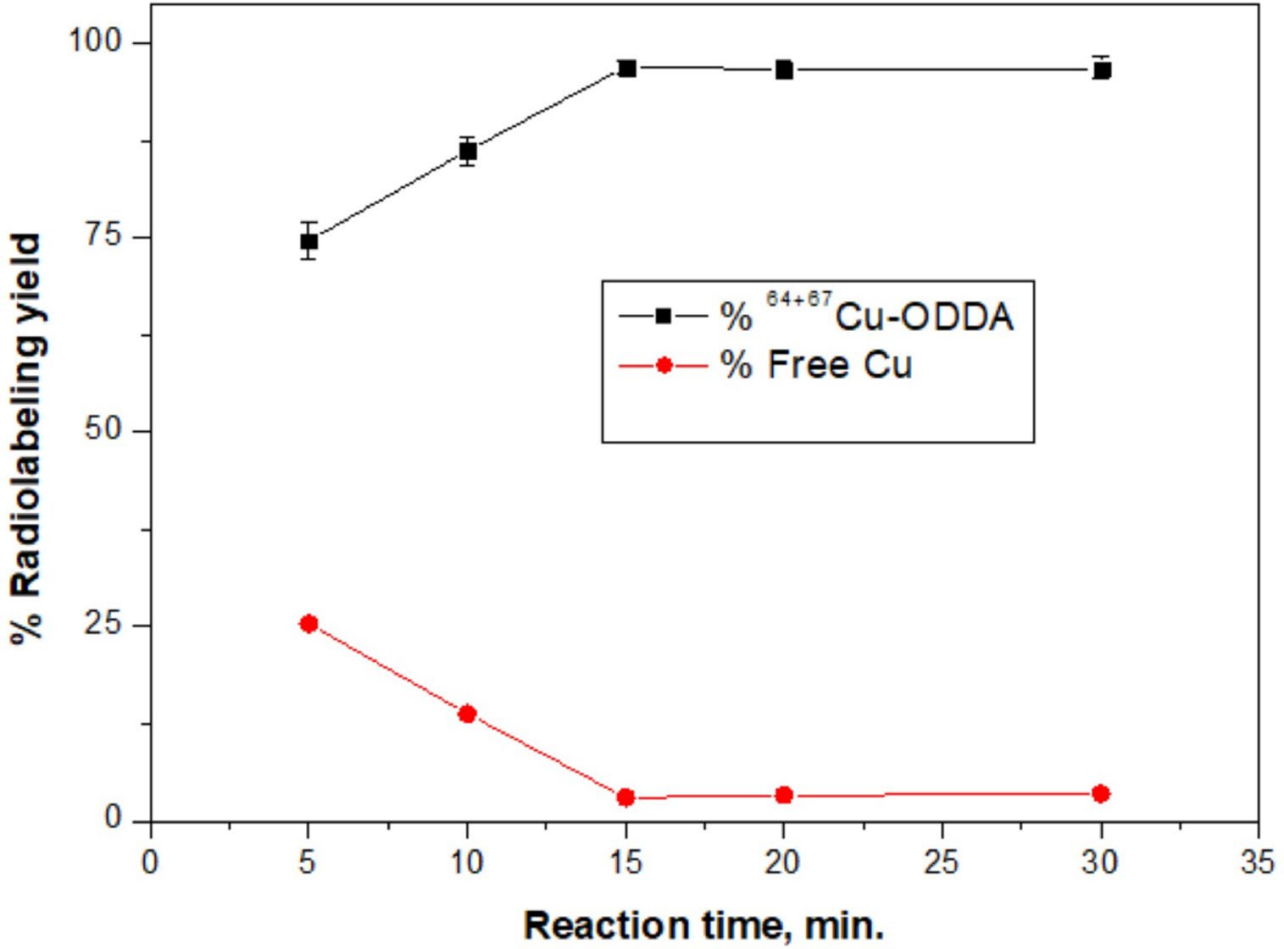
Effect of reaction time on radiolabeling efficiency.

The radiolabeling kinetics were studied at various time intervals ranging from 5 to 30 min. The data (Fig. 4) demonstrate that the labeling yield increased rapidly up to 15 min, reaching 97.0 ± 0.8%. Beyond this time, the efficiency plateaued, suggesting that equilibrium was achieved. Prolonging the reaction didn’t significantly alter the radiolabeling yield.

#### Effect of temperature

**Fig. 5:**
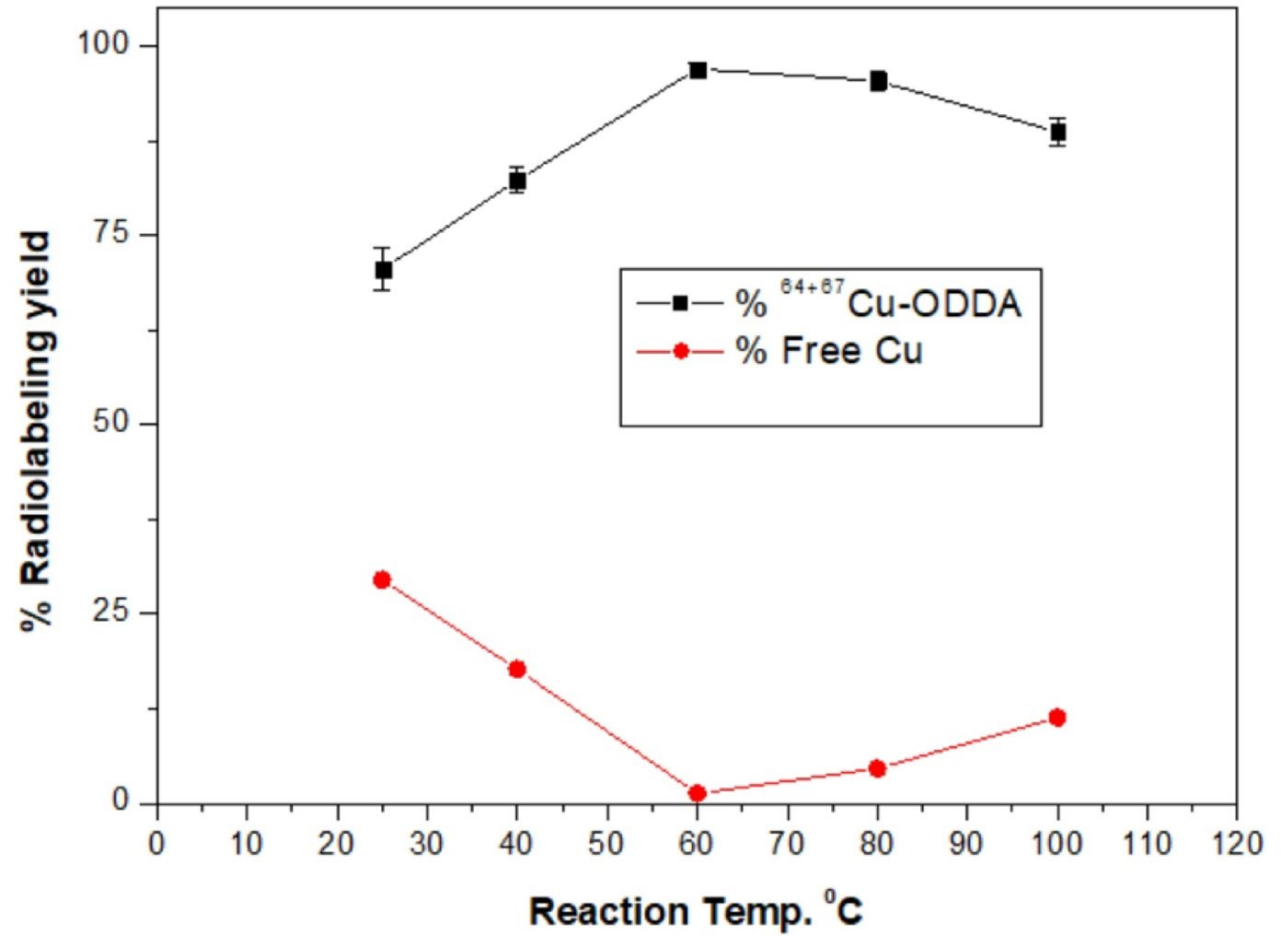
Effect of temperature on radiolabeling efficiency.

Temperature plays a crucial role in accelerating radiolabeling reactions by enhancing ligand–metal complexation kinetics. The radiolabeling efficiency was assessed at temperatures ranging from 25 to 100 °C. The highest radiolabeling yield (97.0 ± 0.8%) was obtained at 60 °C (Fig. 5). At lower temperatures (< 60 °C), incomplete complexation led to lower labeling yields, whereas higher temperatures (> 60 °C) caused ligand instability, possibly due to thermal degradation.

### In-vitro stability of ^64+67^Cu-ODDA complex

The in-vitro serum stability of the ^64+67^Cu-ODDA complex was evaluated over a five-day period and the results are presented in Table 1. The data clearly illustrates that the complex remained stable throughout the study, with minimal dissociation observed. This high stability is indicative of a robust ligand–metal coordination environment. Furthermore, the consistent radiochemical yield over the five-day incubation period suggests that the complex is resistant to transchelation and degradation in serum. This characteristic is crucial for in-vivo applications, as it ensures that the radiolabeled compound remains intact during circulation, thereby improving targeting efficacy and reducing potential toxicity from free radionuclides.

**Table 1:** In-Vitro serum stability of the ^64+67^Cu-ODDA complex over five days.

Time point (Days)	Radiolabeling yield (%) ± SD
0	97.0 ± 0.8
1	97.0 ± 0.3
2	96.9 ± 0.7
3	96.8 ± 0.2
4	96.8 ± 0.4
5	96.6 ± 0.1

### Lipophilicity assessment of the ^64+67^Cu-ODDA complex

The log P value of the ^64+67^Cu-ODDA complex was determined to be -0.8, indicating a hydrophilic nature. This result can be explained by examining the chemical structure of the ligand and its interaction with Cu(II) ions. The presence of oxadiazole rings within the molecular framework introduces polar heteroatoms (oxygen and nitrogen), which significantly enhance hydrogen bonding interactions with water molecules, reducing lipophilicity. Additionally, the aniline groups contribute to increased solubility in aqueous environments due to their hydrophilic amine functionality, further favoring a negative log P value. Furthermore, Copper(II) coordination typically enhances polarity due to strong metal–ligand charge interactions, which further reduces the overall lipophilicity of the complex.

### Biodistribution profile of the ^64+67^Cu-ODDA complex in solid tumor bearing mice

The in-vivo biodistribution of the ⁶^4^⁺⁶⁷Cu-ODDA complex was evaluated in Ehrlich ascites carcinoma-bearing mice at multiple time points, and the results are summarized in Table 2. The radiotracer demonstrated an initial tumor accumulation of 7.8 ± 0.4% ID/g at 0.5 h p.i., which rose markedly to a peak value of 18.0 ± 0.3% ID/g at 2 h. This suggests rapid and efficient early tumor localization, likely driven by enhanced permeability and retention (EPR) effects and favorable pharmacokinetics. However, the subsequent decline in tumor uptake to 2.1 ± 0.2% ID/g at 24 h indicates limited retention, possibly due to insufficient intracellular internalization or rapid systemic redistribution, both common limitations for low molecular weight, hydrophilic tracers. Despite this decline, tumor-to-muscle (T/M = 7.0) and tumor-to-blood (T/B = 2.1) ratios at 24 h remained above acceptable thresholds, suggesting retained imaging contrast and potential for short-term therapeutic intervention, particularly for isotopes with lower therapeutic dose requirements.

**Table 2:** Biodistribution of the ^64+67^Cu-ODDA complex in tumor-bearing mice at various time points.

Organ/tissue	0.5 h p.i	1 h p.i	2 h p.i	4 h p.i	12 h p.i	24 h p.i
Tumor (Right Thigh)	7.8 ± 0.4	13.5 ± 1.1	18.0 ± 0.3	7.8 ± 0.7	4.5 ± 0.4	2.1 ± 0.2
Left Muscle (Control Thigh)	1.4 ± 0.2	2.2 ± 0.3	1.9 ± 0.2	0.9 ± 0.2	0.6 ± 0.3	0.3 ± 0.1
Liver	6.0 ± 0.7	5.5 ± 0.6	4.8 ± 0.5	3.9 ± 0.4	2.8 ± 0.3	1.9 ± 0.2
Kidneys	10.5 ± 0.8	11.2 ± 0.7	10.8 ± 0.6	9.6 ± 0.5	7.4 ± 0.4	5.1 ± 0.3
Lungs	5.8 ± 0.5	4.9 ± 0.4	4.0 ± 0.4	3.1 ± 0.3	2.2 ± 0.2	1.5 ± 0.2
Spleen	2.4 ± 0.3	2.2 ± 0.3	2.0 ± 0.2	1.7 ± 0.2	1.2 ± 0.2	0.8 ± 0.1
Heart	2.0 ± 0.3	1.8 ± 0.3	1.6 ± 0.2	1.3 ± 0.2	1.0 ± 0.2	0.6 ± 0.1
Bone	2.5 ± 0.3	2.3 ± 0.2	2.0 ± 0.2	1.6 ± 0.2	1.3 ± 0.2	1.0 ± 0.2
Intestine	2.8 ± 0.4	2.5 ± 0.3	2.2 ± 0.3	1.9 ± 0.2	1.4 ± 0.2	0.9 ± 0.1
Blood	4.0 ± 0.5	3.5 ± 0.4	3.0 ± 0.3	2.4 ± 0.3	1.8 ± 0.2	1.0 ± 0.2
T/NT	5.6	6.1	9.4	8.6	7.5	7.0
Tumor/Blood	1.9	3.9	6.0	3.3	2.5	2.1

Renal uptake was notably high, starting at 10.5 ± 0.8% ID/g at 0.5 h and persisting at 5.1 ± 0.3% ID/g at 24 h. This reflects the compound’s hydrophilic profile (log P = –0.8) and supports renal filtration as the predominant clearance route. The residual renal activity likely results from partial tubular reabsorption or slow excretory kinetics. Importantly, such renal accumulation is expected to be mitigated in clinical translation via saline hydration or forced diuresis protocols.

Liver uptake exhibited a consistent decline from 6.0 ± 0.7% at 0.5 h to 1.9 ± 0.2% at 24 h, indicating minimal hepatobiliary excretion and reduced nonspecific protein binding. Uptake in non-target organs such as the lungs, spleen, and heart remained low throughout the study, suggesting limited off-target deposition and a clean biodistribution background.

In summary, while long-term tumor retention requires further optimization, the early tumor uptake, favorable T/NT ratios, and rapid renal clearance collectively highlight the diagnostic value of the ⁶^4^⁺⁶⁷Cu-ODDA complex. These findings also provide a sound foundation for developing structurally optimized analogs with enhanced therapeutic efficacy.

### Docking study of ODDA ligand

**Fig. 6:**
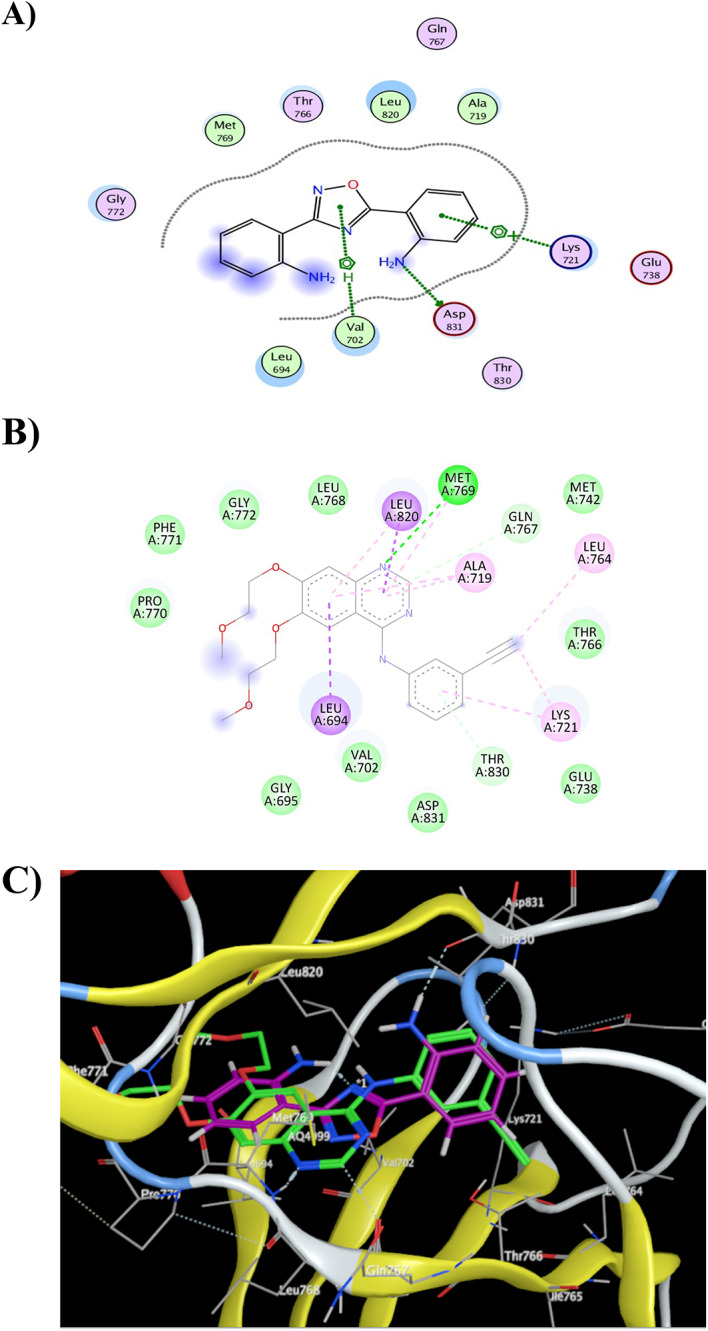
Interaction of 2,2'-(1,2,4-oxadiazole-3,5-diyl) di aniline with EGFR (PDB ID: 1M17). (**A**) 2D interaction diagram showing dianiline (magenta) compared with the co-crystallized ligand erlotinib (green), with docking scores of −12.525 for dianiline and −12.309 for erlotinib (RMSD = 1.93). (**B**) 2D bond-length map of the co-crystallized ligand erlotinib with selected residues, including Val (4.15 Å), Asp (2.14 Å), and Lys (3.98 Å). (**C**) 3D docking pose of 2,2'-(1,2,4-oxadiazole-3,5-diyl) di aniline within the EGFR binding pocket.

**Fig. 7:**
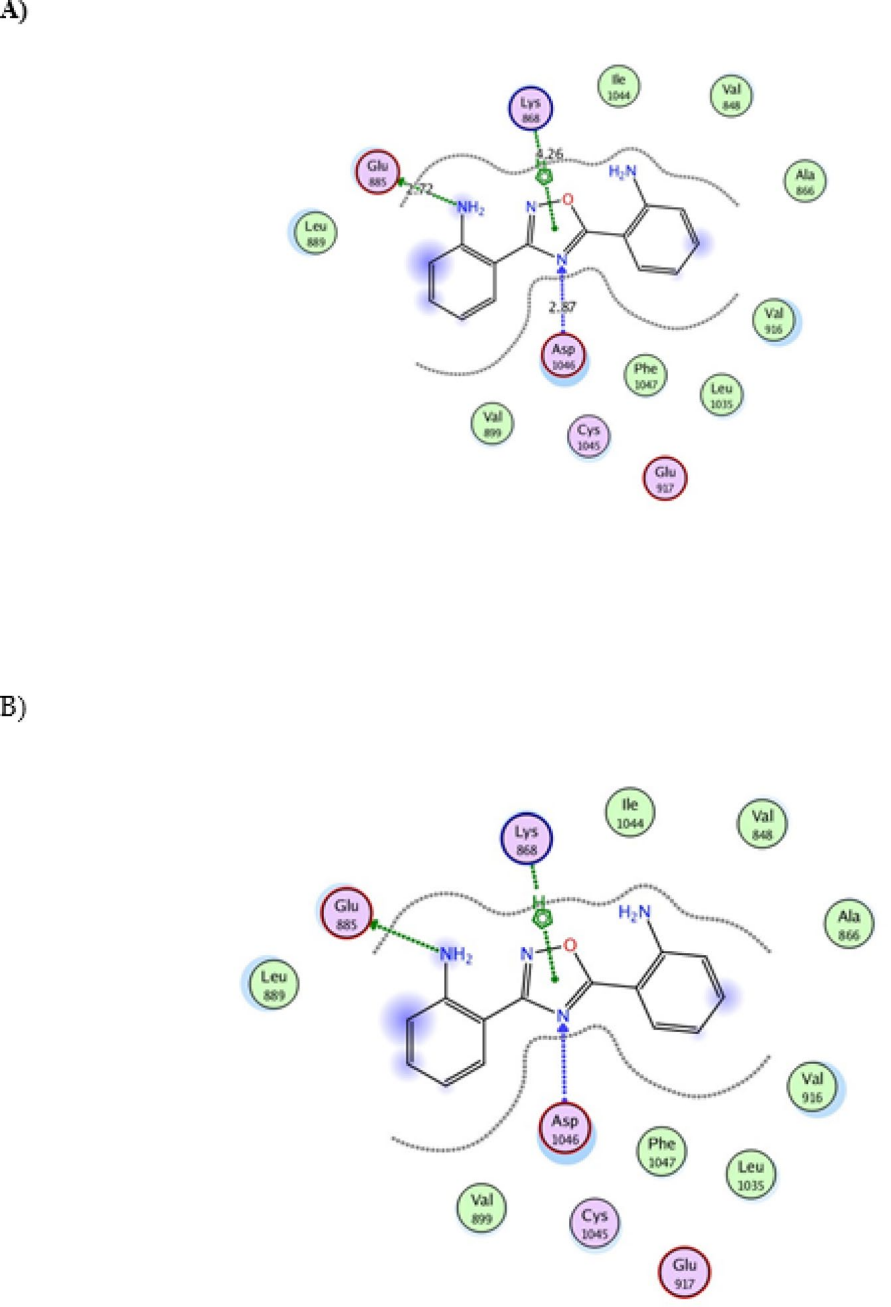

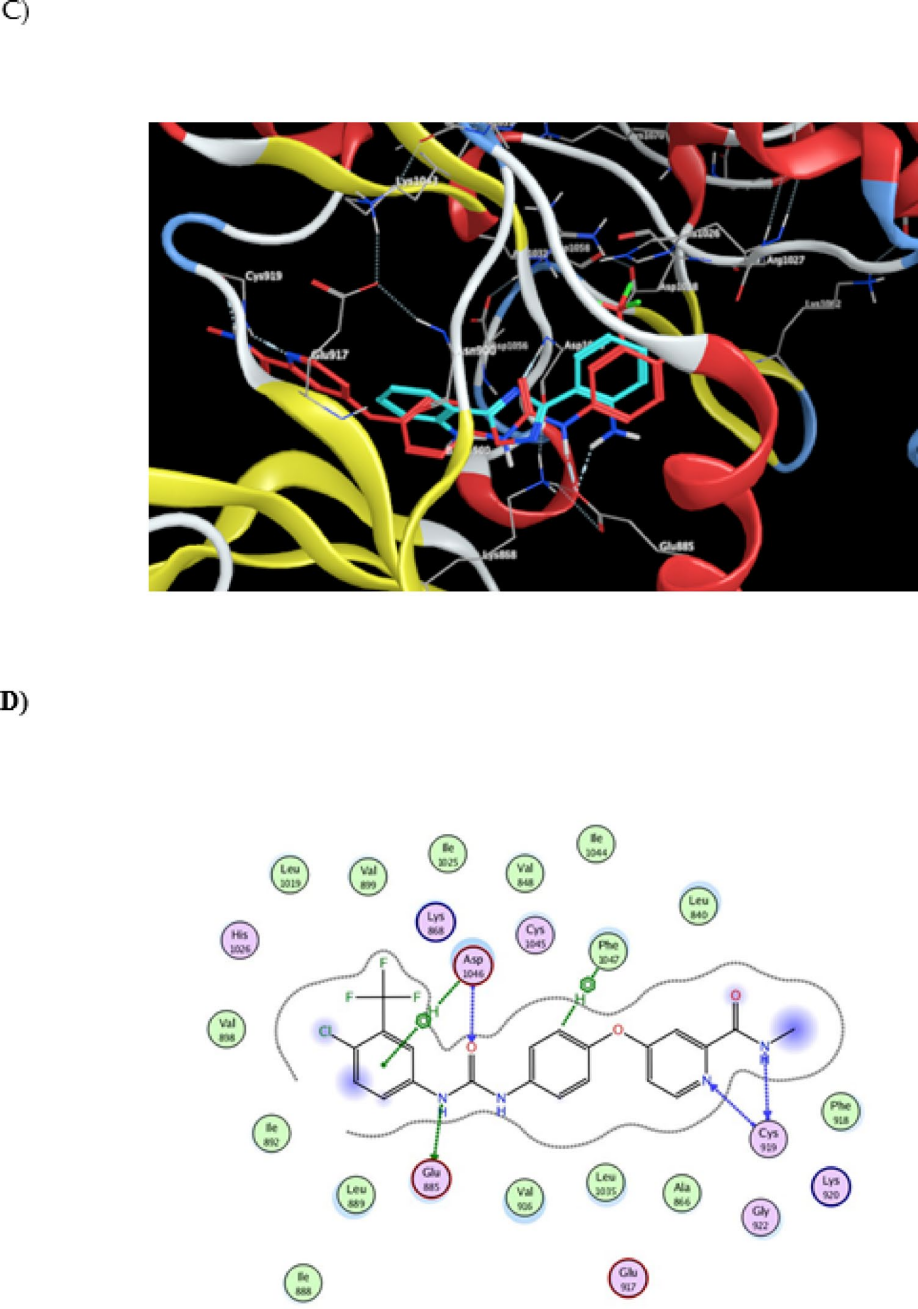
Interaction of 2,2'-(1,2,4-oxadiazole-3,5-diyl) di aniline with VEGFR2 (PDB ID: 4ASD). (**A**) 2D interaction diagram of dianiline (turquoise). (**B**) 2D interaction diagram of the co-crystallized ligand sorafenib (red), with docking scores of −16.243 for dianiline and −17.105 for sorafenib (RMSD = 1.87). (**C**) 3D docking pose of 2,2'-(1,2,4-oxadiazole-3,5-diyl) di aniline within the VEGFR2 binding pocket. (**D**) Chemical structure of the co-crystallized ligand sorafenib.

The anticancer potential of 2,2'-(1,2,4-oxadiazole-3,5-diyl) di aniline was evaluated through molecular docking studies. The compound was docked as the guest ligand against two angiogenesis-related receptor proteins, epidermal growth factor receptor (EGFR) (Fig. 6) and vascular endothelial growth factor receptor 2 (VEGFR2) (Fig. 7), using AutoDock analysis to predict binding affinity and interaction profiles.

Our findings indicate that ODDA ligand binds to proliferative angiogenic receptors and modulates their activity^45–47^. This suggests that the compound acts as both an antiproliferative and antiangiogenic agent across various cancer types^48–50^, with particular relevance for highly chemoresistant forms such as triple-negative breast cancer (TNBC).

### Docking study of ^64+67^Cu-ODDA complex

**Fig. 8:**
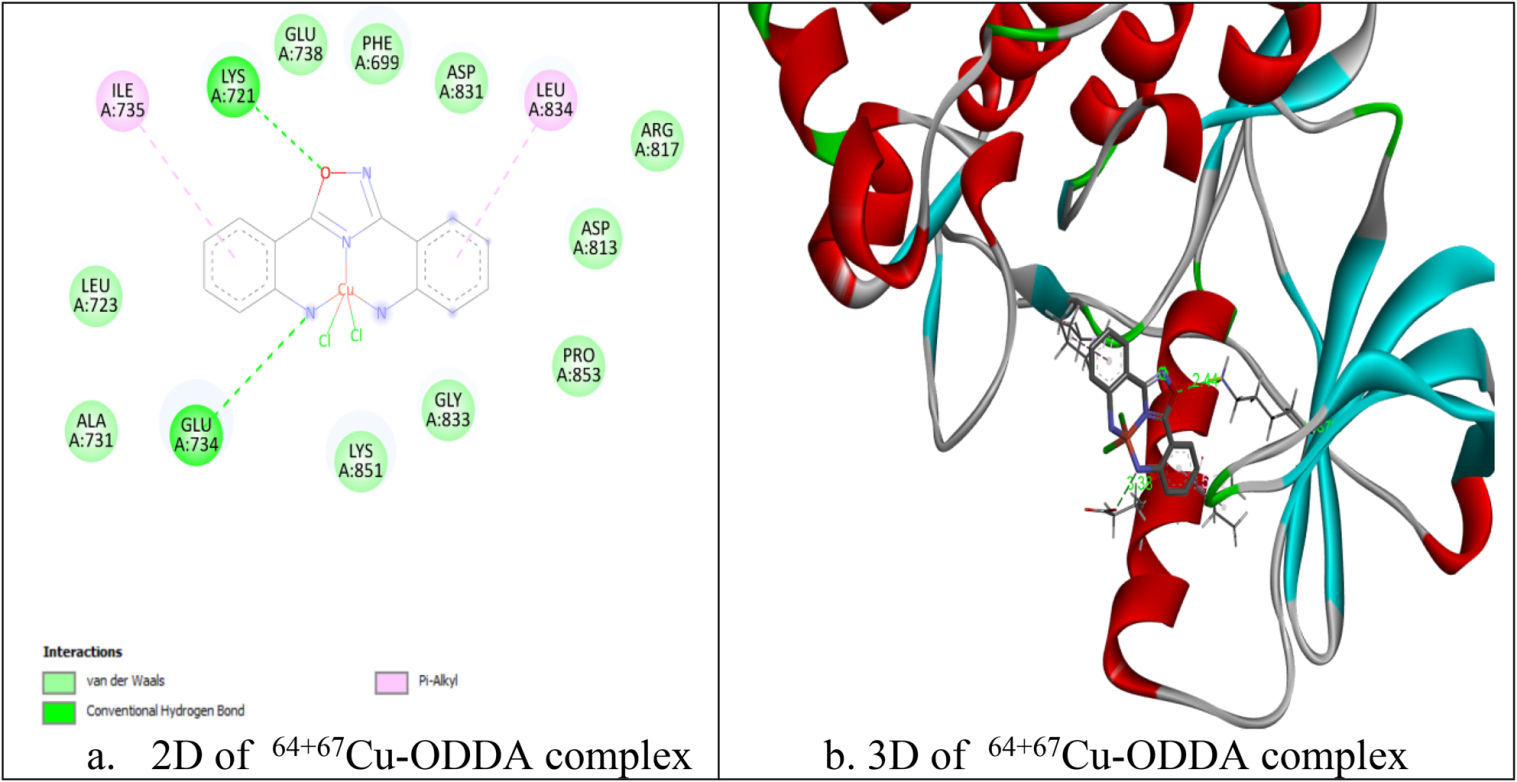
Interaction of ^64+67^Cu-ODDA complex with EGFR ( PDB ID 1M17).

**Fig. 9:**
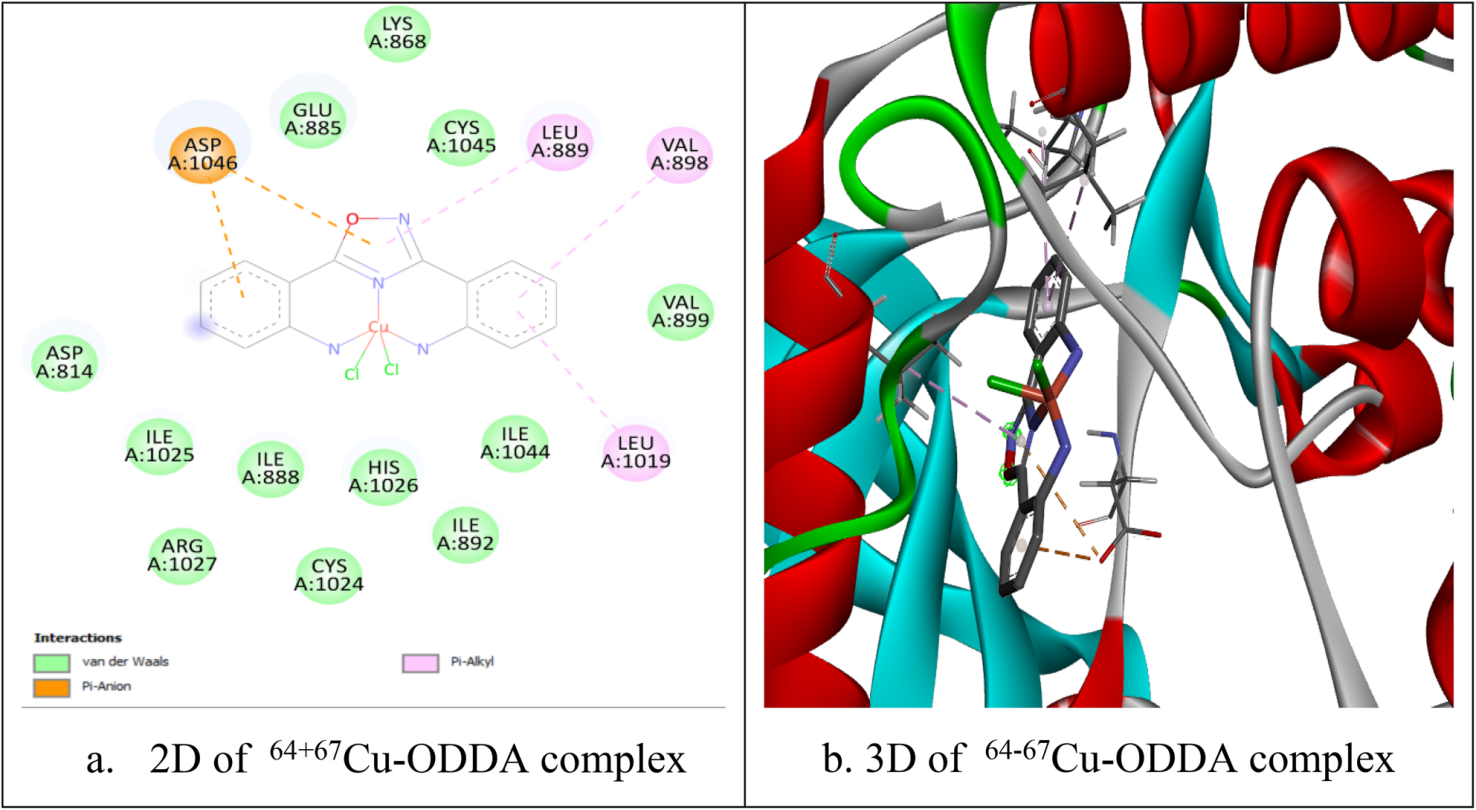
Interaction of ^64+67^Cu-ODDA complex with VEGFR2 (PDB ID 4ASD).

The complex was able to accommodate within the active site of EGFR kinase domain and could form number of interactions that stabilize the complex within the pocket of the enzyme, making it as candidate inhibitor (Fig. 8). For example it could form van der Waals interactions and pi interactions (pi-alky). The binding energy was -9.1 kcal/mol. It also could form two hydrogen bonds with residues lys 721 and glu734 (bond lengths: 2.44 & 3.38Å), which contributes to more stabilization of the complex within enzyme pocket helping it in its proposed action. As shown in Fig. 9, when the complex was docked within VEGFR2 kinase domain it could be well accommodated within the pocket of the enzyme forming number of interactions (van der Waals interactions and different types of pi interactions; pi-alky and pi-pi) similar to those formed by the co-crystalized inhibitor (Sorafenib), as in Asp1046, leu889, leu1019 with binding energy -8.6 kcal/mol.

## Conclusion

The ⁶^4^⁺⁶⁷Cu-ODDA complex was successfully radiolabeled with high radiochemical purity and in-vitro stability, confirming its potential as a theranostic agent for solid tumors. The biodistribution profile demonstrated significant tumor uptake, peaking at 18.0% ID/g at 2 h post-injection, along with rapid renal clearance, consistent with its hydrophilic nature (log P = -0.8). Molecular docking results suggest that ^64+67^Cu-ODDA may interact with EGFR and VEGFR2, potentially contributing to anticancer activity. While current data emphasize diagnostic utility, the complex may still hold therapeutic promise following further optimization of its pharmacokinetic properties.

## Data Availability

All data supporting the findings of this study are included within the manuscript. Additional datasets are available from the corresponding author upon reasonable request.
